# Expression of Senescence-Associated microRNAs and Target Genes in Cellular Aging and Modulation by Tocotrienol-Rich Fraction

**DOI:** 10.1155/2014/725929

**Published:** 2014-07-14

**Authors:** Sharon Gwee Sian Khee, Yasmin Anum Mohd Yusof, Suzana Makpol

**Affiliations:** Department of Biochemistry, Faculty of Medicine, Universiti Kebangsaan Malaysia, Jalan Raja Muda Abdul Aziz, 50300 Kuala Lumpur, Malaysia

## Abstract

Emerging evidences highlight the implication of microRNAs as a posttranscriptional regulator in aging. Several senescence-associated microRNAs (SA-miRNAs) are found to be differentially expressed during cellular senescence. However, the role of dietary compounds on SA-miRNAs remains elusive. This study aimed to elucidate the modulatory role of tocotrienol-rich fraction (TRF) on SA-miRNAs (miR-20a, miR-24, miR-34a, miR-106a, and miR-449a) and established target genes of miR-34a (CCND1, CDK4, and SIRT1) during replicative senescence of human diploid fibroblasts (HDFs). Primary cultures of HDFs at young and senescent were incubated with TRF at 0.5 mg/mL. Taqman microRNA assay showed significant upregulation of miR-24 and miR-34a and downregulation of miR-20a and miR-449a in senescent HDFs (*P* < 0.05). TRF reduced miR-34a expression in senescent HDFs and increased miR-20a expression in young HDFs and increased miR-449a expression in both young and senescent HDFs. Our results also demonstrated that ectopic expression of miR-34a reduced the expression of CDK4 significantly (*P* < 0.05). TRF inhibited miR-34a expression thus relieved its inhibition on CDK4 gene expression. No significant change was observed on the expression of CCND1, SIRT1, and miR-34a upstream transcriptional regulator, TP53. In conclusion tocotrienol-rich fraction prevented cellular senescence of human diploid fibroblasts via modulation of SA-miRNAs and target genes expression.

## 1. Introduction

Tocotrienols, the lesser known isomer of vitamin E, have gained increasing scientific interest in the study of aging and aging-related diseases due to its eminent antioxidant effects and nonantioxidant activity [1]. Palm oil is one of the richest natural sources of tocotrienol. Tocotrienol extracted from palm oil consists mainly of *α*-tocopherol and a mixture of four tocotrienol isomers (*α*, *β*, *γ*, and *δ*), referred to as tocotrienol-rich fraction (TRF) [2].

Accumulating evidences demonstrated that tocotrienol modulates several mechanisms associated with aging. In individuals over 50 years old, tocotrienol-rich fraction supplementation decreased DNA damage [3] and reduced the level of advanced glycosylation end products (AGE) and protein carbonyls, which are the oxidative damage indicators during aging [4]. In animal model of aging, tocotrienol extended mean lifespan by reducing protein carbonylation [5]. In replicative cell aging model, tocotrienol-rich fraction reversed cellular aging by preventing cell cycle arrest while restoring telomere length [6].

Human diploid fibroblasts (HDFs) undergo irreversible proliferative arrest, termed as replicative senescence, after around 50 cell divisions when cultured* in vitro*. This makes HDFs a suitable experimental model in the study of cellular aging [7]. Permanent arrest of proliferation accompanied by striking changes in cellular phenotype is the hallmark of cellular senescence. Deposition of senescent cells with age disrupts the normal tissue structure and function, further suggesting a relationship between senescence and aging [8].

Proliferating cells succumbed to cell cycle arrest when cellular macromolecules (DNA, protein, and lipid) are damaged by reactive oxygen species (ROS) constantly generated during physiological metabolism [9]. Besides the free radical theory of aging, various signal transduction pathways that regulate aging have been proposed, including insulin/IGF-1 signalling, TOR signalling, AMPK, and sirtuins [10].

The role of miRNAs in regulating aging process has been established recently, with the discovery of miRNA, lin-4 that regulates the lifespan in* Caenorhabditis elegans* [11]. Since then, various studies have characterized numerous microRNAs (miRNAs) that are differentially expressed during aging at cell, tissue, and organism levels. Individual miRNAs contribute to accelerate or decelerate aging by targeting components of conserved aging signalling pathways [12]. These small and noncoding RNAs (≈22 nucleotides) regulate gene expression at posttranscriptional level by binding to its target mRNA mainly at the 3′ untranslated region (3′-UTR). The binding may inhibit protein translation or result in mRNA degradation [13].

Several miRNAs (including miR-20a, miR-24, miR-34a, miR-106a, and miR-449a) that funnel proliferating cells to senescence regulate cellular senescence via either or both p53/p21 and p16/pRb pathways [14]. The coordinated action between SA-miRNAs in p53/p21 and p16/pRb pathway with transcription factors (Myc and E2F) in cell cycle regulation contributes to the inhibition of cell proliferation during cellular senescence [15]. The SA-miRNAs control cell transition, mainly through the G_1_/S checkpoint during cell cycle progression by targeting the components of cell cycle including cyclin-dependent kinases (CDKs) and cyclin-dependent kinase inhibitors (CDKIs) [16].

Despite the reported discrepancies between the upregulation and downregulation of miRNAs during aging and cellular senescence, such as miR-34a [17, 18], study of the modulatory effect of dietary compound on miRNAs may aid in the understanding of how SA-miRNAs can be regulated in favour of slowing down aging process or reducing aging phenotypes. Modulation of miRNAs by dietary and pharmacological agents has been reported recently [19]. In view of this, the present study was designed to evaluate the possible modulatory role of tocotrienol-rich fraction on the expression of SA-miRNAs and their target genes, which could potentially be exploited for reversing cellular aging.

The present study aimed to elucidate the molecular mechanism of TRF in reversing cellular aging through cell cycle arrest prevention focussing on the modulation of SA-miRNAs expression and, hence, alteration of their target genes expression which are involved in cell cycle regulation.

## 2. Materials and Methods

### 2.1. Sample Collection

This research was conducted with the approval of Ethics Committee of Universiti Kebangsaan Malaysia (Approval Project Code: FF-215-2013). Primary HDFs were derived from circumcised foreskins of 9 to12 year-old boys. Written consents were obtained from parents of all subjects.

### 2.2. Cell Culture and Serial Passaging

Aseptically collected skin samples were rinsed several times with 75% alcohol and phosphate buffered saline (PBS) containing 1% antibiotic-antimycotic solution (PAA, Austria). After removing the epidermis, the dermis was cut into small pieces and transferred into 0.03% collagenase type I digestive buffer (Worthington Biochemical Corporation, USA). Pure dermis was digested in incubator shaker at 37°C for 6–12 h. The isolated cells were rinsed with PBS before being cultured in Dulbecco's modified Eagle's medium (DMEM) containing 10% foetal bovine serum (FBS) (PAA, Austria) and 1% antibiotic-antimycotic solution at 37°C in 5% CO_2_ humidified incubator. After 5-6 days, the cultured HDFs were trypsinized and culture-expanded into new T25 culture flasks. When the subcultures were 80–90% confluent, serial passaging was done by trypsinization while the number of population doublings (PDs) was monitored until HDFs reached senescence. For subsequent experiments, HDFs used were at passage 6 (young HDFs, PD < 12) and passage 30 (senescent HDFs, PD > 55).

### 2.3. TRF Preparation and Treatment

Stock solution of TRF was freshly prepared in dark by dissolving 1 g Gold Tri E 50 (Sime Darby Bioganic Sdn. Bhd., Malaysia) in 1 mL 100% ethanol (1 : 1) and kept at −20°C for not more than one month. TRF was activated by incubating 45 *μ*L stock TRF (1 g/1 mL) with 60 *μ*L FBS overnight at 37°C. To prepare TRF at 50 mg/mL, 90 *μ*L DMEM with 10% FBS and 105 *μ*L 100% ethanol were added to the activated TRF, after which 600 *μ*L mixture containing FBS and 100% ethanol (1 : 1) was also added. TRF at 0.5 mg/mL was prepared in culture medium by mixing 5 *μ*L TRF (50 mg/mL) and 495 *μ*L DMEM with 10% FBS. Cells were plated at 2 × 10^4^ in 24-well plate and incubated overnight. Treated groups were incubated with 0.5 mg/mL TRF for 24 h; untreated HDFs were incubated with DMEM containing 10% FBS (PAA, Austria) while transfected untreated HDFs were incubated with DMEM containing 5% FBS (PAA, Austria) without antibiotic. Media for untreated cells were changed parallel to the treated cells and both were harvested on the same day.

### 2.4. Morphological Analysis and Senescence-Associated Beta-Galactosidase (SA-*β*-gal) Staining

SA-*β*-gal staining was performed with a senescent cells staining kit (Sigma, USA) according to the manufacturer's instructions. Blue staining was visible after 4 h of incubation with *β*-galactosidase staining solution containing 5-bromo-4-chloro-3-indolyl-*β*-D-galactosidase (X-gal) at 37°C in the absence of CO_2_.

### 2.5. Primer Design

Forward primers for miRNAs were designed according to the miRNAs sequences listed in miRBase database (http://www.mirbase.org).  Table 1 shows the forward primer sequences for validated miRNAs. Primers for human GAPDH, CCND1, CDK4, SIRT1, and TP53 were designed from listed NIH GenBank database using Primer 3 software and blasted with GenBank database sequences for specificity confirmation. The efficiency and specificity of each primer set were confirmed via standard curve (Ct value versus serial dilution of total RNA) and melting profile evaluation. The primers sequences for quantitative gene expression analysis are shown in Table 2.

### 2.6. RNA Extraction

Total RNA was extracted from different groups of HDFs using TRI Reagent (Molecular Research Center, Cincinnati, USA) according to the manufacturer's instructions. Polyacryl Carrier (Molecular Research Center, Cincinnati, USA) was added to each extraction to precipitate the total RNA. Extracted RNA pellet was washed with 75% ethanol and dried prior to dissolving it in RNase-free and DNase-free distilled water. Aliquots of total RNA were stored at −80°C immediately after extraction. The yield and purity of extracted total RNA were determined by Nanodrop (Thermo Scientific, USA).

### 2.7. Transfection

Young HDFs were reverse transfected with mirVana miR-34a Mimic 1 (Ambion, USA) at a final concentration of 10 nM to overexpress miR-34a in the cells, using Lipofectamine RNAiMAX (Invitrogen, USA). 3 × 10^4^ cells were plated and transfected in DMEM containing 2% FBS (PAA, Austria) without antibiotic for 24 h. mirVana miRNA mimic and Negative Control #1 (Ambion, USA) were used as control. Total RNA was extracted from nontreated and TRF treated transfected cells after treatment for another 24 h.

### 2.8. Real Time qRT-PCR

For quantitative analysis of miRNAs, reverse transcription (RT) was first performed using Taqman MicroRNA Reverse Transcription kit (Applied Biosystems, USA) according to manufacturer's instructions with total RNA at 10 ng. PCR reactions were then performed according to manufacturer's instructions to quantitate the expression levels of miRNAs (miR-20a, miR-24, miR-34a, miR-106a, and miR-449a) using Taqman Universal PCR Master Mix, No AmpErase UNG (Applied Biosystems, USA), and Taqman microRNA assay (Applied Biosystems, USA) for the miRNAs of interest. The PCR amplification was performed in iQ5 Multicolor Real Time PCR (Bio Rad, USA) at 95°C for 10 min, followed by 40 cycles of 95°C for 15 s and 60°C for 60 s. The PCR incubation profile was extended to 45 cycles for miR-20a and miR-449a. PCR reactions were performed in triplicate. All miRNAs expressions were normalized to the expression of RNU6B. The relative expression value (REV) of miRNAs was calculated using the 2^−ΔCt^ method of relative quantification [20] as the equation

(1) $$\begin{array}{c} \text{REV}=2^{\text{Ct}\;\text{value}\;\text{of}\;\text{RNU}6\text{B}-\text{Ct}\;\text{value}\;\text{of}\;\text{miRNA}}. \end{array}$$

Gene expression levels of CCND1, CDK4, SIRT1, and TP53 were analysed with KAPA SYBR Fast 1-Step qRT-PCR kit and Bio-Rad iCycler (KAPA Biosystems, USA). Each qRT-PCR mixture contained 11.7 *μ*L nuclease free water, 10 *μ*L KAPA SYBR Fast master mix, 0.3 *μ*L RT enzyme, 1 *μ*L 100 *μ*M forward primer, 1 *μ*L 100 *μ*M reverse primer, and 1 *μ*L total RNA at 50–100 ng. Reactions were performed in iQ5 Multicolor Real Time PCR (Bio Rad, USA) at 42°C for 5 min and 95°C for 4 min, followed by 40 cycles of 95°C for 3 s and 60°C for 20 s. qRT-PCR reactions were performed in duplicate. GAPDH was used as a reference gene in gene expression normalization [21]. The relative expression value (REV) of genes of interest was calculated using the 2^−ΔCt^ method of relative quantification [22] as the equation

(2) $$\begin{array}{c} \text{REV}=2^{\text{Ct}\;\text{value}\;\text{of}\;\text{GAPDH}-\text{Ct}\;\text{value}\;\text{gene}\;\text{of}\;\text{interest}}. \end{array}$$

### 2.9. Statistical Analysis

Data were presented as mean ± SD. ANOVA was used for multiple comparisons of groups. Mann-Whitney *U* test was used to assess statistical significance between groups. A value of *P* < 0.05 was considered statistically significant.

## 3. Results

### 3.1. Morphological Analysis and SA-*β*-Galactosidase Staining

**Figure 1:**
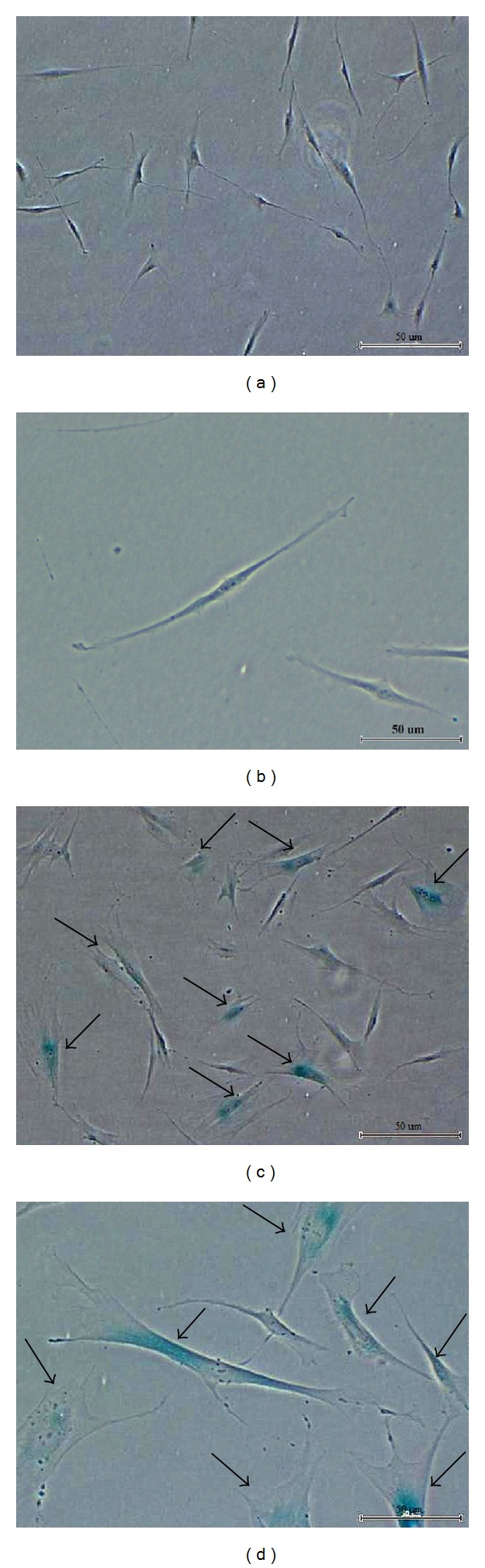
Morphological changes and SA-*β*-gal staining of young HDFs (magnification 40x) (a), young HDFs (magnification 100x) (b), senescent HDFs (magnification 40x) (c), and senescent HDFs (magnification 100x) (d). Senescent HDFs showed morphological changes during replicative senescence with the loss of original fibroblastic shape, appearance of flattened morphology, and increased in the size of cells and nucleus. Only senescent HDFs showed positive SA-*β*-gal staining in blue as indicated by arrow.

Changes in cell morphology and increase in SA-*β*-gal activity were characterized as aging phenotypes. Young HDFs displayed the normal spindle shape of a typical fibroblast and were not stained blue in SA-*β*-gal staining (Figures 1(a) and 1(b)). However, senescent HDFs were mainly characterized by cellular enlargement and flattening with a concomitant increase in the size of nucleus. Positive blue stain of SA-*β*-gal staining mainly appeared in HDFs at passage 30 suggesting that HDFs at this passage had reached senescence (Figures 1(c) and 1(d)).

### 3.2. TRF Treatment Modulates the Expression of SA-miRNAs

**Figure 2:**
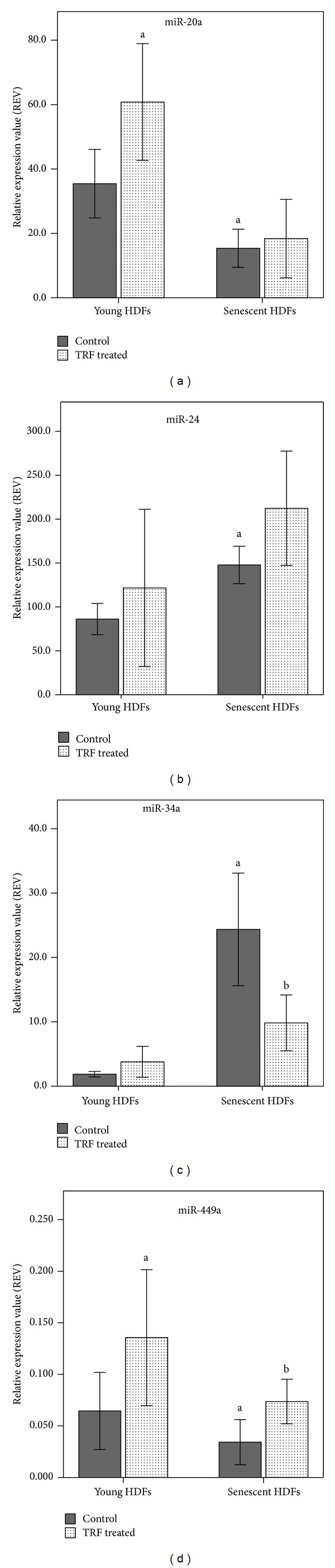
Effect of TRF treatment on the expression levels of miR-20a (a), miR-24 (b), miR-34a (c), and miR-449a (d) in young and senescent HDFs measured by real time qRT-PCR. ^a^denotes  *P* < 0.05 compared to control young HDFs and ^b^ *P* < 0.05 compared to control senescent HDFs. Data are presented as relative expression value (REV) normalized to RNU6B expression (mean ± SD, *n* = 9).

Changes in miRNAs expressions were observed in HDFs with senescence. The expression of miR-20a and miR-449a was decreased while the expression of miR-24 and miR-34a was increased significantly in senescent HDFs as compared to young HDFs (*P* < 0.05) (Figure 2). No noticeable level of miR-106a was expressed with trials using 10 ng, 20 ng, and 30 ng total RNA (data not shown). TRF treatment increased miR-20a expression in young HDFs, reduced miR-34a expression in senescent HDFs, and increased miR-449a expression in both young and senescent HDFs (*P* < 0.05). No significant effect was observed on the expression of miR-24 with TRF treatment.

### 3.3. Effect of TRF Treatment on miR-34a Expression in Transfected HDFs

**Figure 3:**
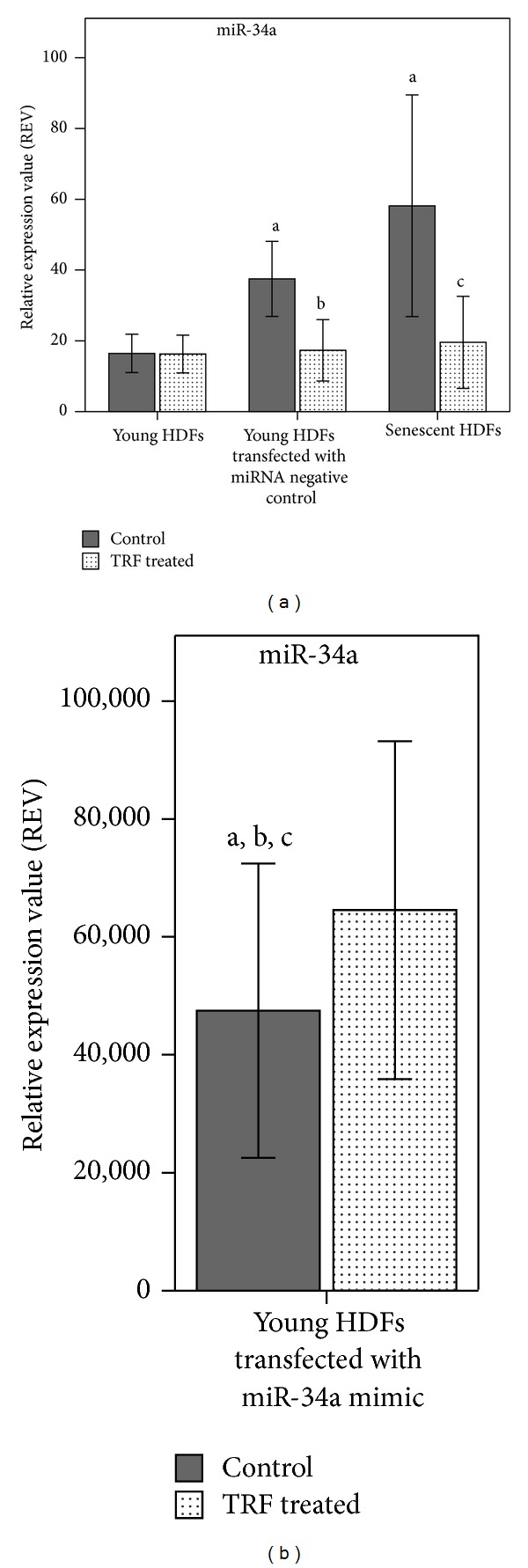
Effect of TRF treatment on the expression level of miR-34a in nontransfected young HDFs, young HDFs transfected with miR-34a mimic, miRNA negative control, and senescent HDFs measured by real time qRT-PCR. Young HDFs were transfected with miR-34a mimic (10 nM) to overexpress miR-34a or miRNA negative control as control for 24 h, followed by TRF treatment for 24 h. ^a^denotes*P* < 0.05 compared to control young untransfected HDFs, ^b^ *P* < 0.05 compared to young HDFs transfected with miRNA negative control, and ^c^ *P* < 0.05 compared to control senescent HDFs. Data are presented as relative expression value (REV) normalized to RNU6B expression (mean ± SD, *n* = 9).

The expression level of miR-34a increased significantly (*P* < 0.05) in young HDFs transfected with miR-34a mimic indicating that transfection process had successfully introduced miR-34a into young HDFs (Figure 3). TRF treatment reduced miR-34a expression significantly in young HDFs transfected with miRNA negative control and senescent HDFs.

### 3.4. TRF Treatment Modulates the Expression of Target Genes and Upstream Regulator of miR-34a

**Figure 4:**
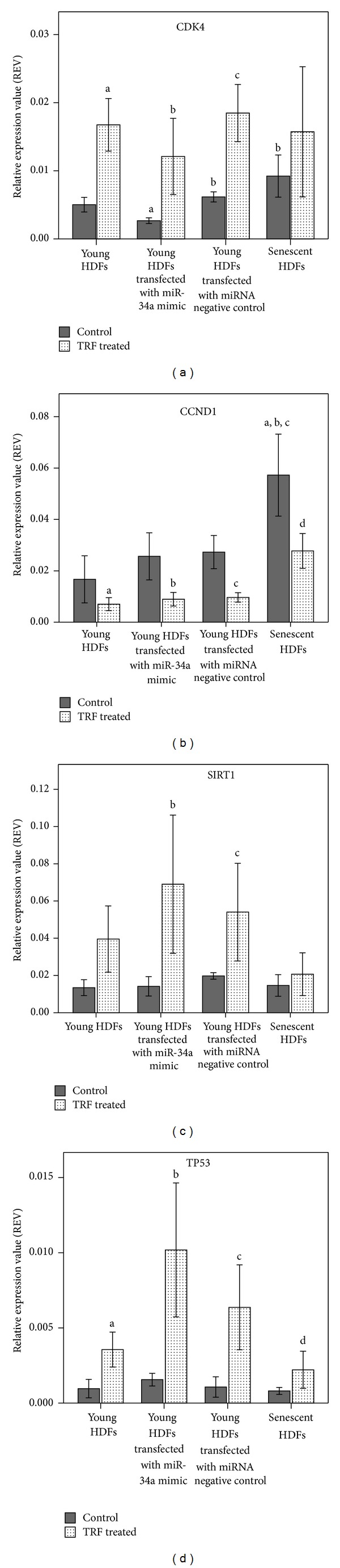
Effect of TRF treatment on the expression level of CDK4 (a), CCND1 (b), SIRT1 (c), and TP53 (d) in nontransfected young HDFs, young HDFs transfected with miR-34a mimic, miRNA negative control, and senescent HDFs measured by real time qRT-PCR. ^a^denotes*P* < 0.05 compared to control young untransfected HDFs, ^b^ *P* < 0.05 compared to control young HDFs transfected with miR-34a mimic, ^c^ *P* < 0.05 compared to control young HDFs transfected with miRNA negative control, and ^d^ *P* < 0.05 compared to control senescent HDFs. Data are presented as relative expression value (REV) normalized to GAPDH expression (mean ± SD, *n* = 6).

Ectopic expression of miR-34a reduced the gene expression of CDK4 significantly (*P* < 0.05), while no significant changes were observed on the gene expression of CCND1 (cyclin D1), SIRT1, and TP53 (Figure 4). TRF treatment was found to increase the expression of CDK4 significantly in young HDFs, young HDFs with ectopic expression of miR-34a, and young HDFs transfected with miRNA negative control (*P* < 005). TRF treatment also reduced CCND1 gene expression in all groups of cells and increased the expression of SIRT1 I in young HDFs with ectopic expression of miR-34a and young HDFs transfected with miRNA negative control (*P* < 0.05). The expression of TP53 increased significantly in all groups of cells treated with TRF (*P* < 0.05).

## 4. Discussion

In this study, cellular morphological changes and increased SA-*β*-gal activity clearly differentiate senescent HDFs from young HDFs. Elevated level of matrix metalloproteinase 1 and decreased level of extracellular matrix components such as elastin and collagen I-1a were believed to have contributed to the shift in the senescent fibroblasts phenotypes towards matrix degradation [23]. Furthermore, increase in the size of HDFs during replicative senescence may be attributed to the increase in size of nucleus and nucleoli and increase in number of vacuoles, Golgi, endoplasmic reticulum, cytoplasmic microfilament, and intracellular vesicles mainly lysosomes [24]. Increased lysosomal content in senescent cells was reflected by the increase of SA-*β*-gal activity [25], widely used as the biomarker to demonstrate the onset of replicative senescence in multiple cell types including human fibroblast cultures [26].

It has been reported that changes in miRNA expression occurred with human aging [18]. Our data on SA-miRNAs expression showed upregulation of miR-24 and miR-34a and downregulation of miR-20a and miR-449a in senescent cells.

Decrease in miR-20a expression in senescent cells observed in this study, which was also reported in previous studies [27, 28], may be attributed to the increase in CCND1 gene expression [29] which was also observed in senescent HDFs. Young HDFs rather than senescent HDFs responded towards TRF treatment, by showing an increase in miR-20a expression. Upregulation of miR-20a in young HDFs after TRF treatment may increase the inhibition effect on p21^Cip1^ [30] and hence increase CDK2 level to form active CDK complexes with cyclin E and cyclin A to promote higher cell proliferation rate in young HDFs [31].

Deep sequencing analysis [17] and loss-of-function analysis [32] supported the upregulation of miR-24 in senescent cells observed in this study despite the contradictory findings that were reported earlier [33, 34]. Increased miR-24 expression in senescent HDFs may inhibit cell proliferation by suppressing cell cycle regulatory genes including E2F2 [32], which then prevent miR-20a promoter activation resulting in decreased miR-20a expression [35]. Interestingly, the expression of one miRNA may affect the other miRNA via its target genes, which is at the same time the transcriptional regulator of the other miRNA. However, TRF treatment did not have any modulatory effect on miR-24 expression in senescent HDFs and also young HDFs.

Increased miR-34a expression in senescent HDFs observed in this study which is in agreement with earlier reported literature [17, 36] may halt cell cycle progression by regulating several components in cell cycle regulation including CCNE2, CDK4 [37], CCND1, and CDK6 [38]. It is intriguing to report that TRF treatment decreased the expression of miR-34a in senescent HDFs. This finding triggered the interest to further study how TRF affects the target genes of miR-34a in reversing cellular aging.

To characterize miR-34a targets, we have identified CDK4, CCND1, and SIRT1 as the target genes of miR-34a, using database of experimentally verified targets of miRNAs (TarBase 6.0) [39] and the bioinformatics miRNA target prediction tools: TargetScan (http://targetscan.org) and microRNA.org (http://www.microRNA.org). CDK4 and CCND1 are involved in cell cycle regulation [40] while SIRT1 is responsive towards oxidative stress which is prominent during aging [41].

Our findings showed that ectopic delivery of miR-34a in young HDFs significantly increased miR-34a expression level which increased the inhibitory effect of miR-34a on target genes. Transfection of miR-34a mimic into young HDFs resulted in sufficient increase in miR-34a levels to cause a corresponding decrease in the expression of the predicted target, CDK4, whereas the gene expression of CCND1, SIRT1, and TP53 was not affected.

Elevated level of miR-34a in senescent HDFs was not sufficient to repress CDK4 gene expression. However, ectopic expression of miR-34a showed significant inhibition effect on CDK4 gene expression, suggesting that miR-34a level is important in determining its effect on CDK4 gene expression. TRF treatment increased CDK4 gene expression in young nontransfected and transfected HDFs but not senescent HDFs. This interestingly suggested that TRF treatment suppressed miR-34a expression and thus relieved its inhibition on CDK4 gene expression. Increased CDK4 level encourages more cyclin D1/CDK4/CDK6 complexes to be formed, which favours cell cycle progression and cell proliferation. In addition, high level of CDK4 ensures its function will not be diminished completely by p16^INK4a^ [31, 34].

Decreased CCND1 gene expression was reported with ectopic expression of miR-34a with a higher concentration of miR-34a duplex (50 nM) [38]. Increased CCND1 gene expression in senescent HDFs was observed in this study, in accordance with previously reported data [23]. CCND1 mostly formed inactive CDK complex with inactive unphosphorylated CDK2 [42]. TRF treatment decreased CCND1 gene expression directly in young and senescent HDFs regardless of miR-34a modulation. One of the isomer of TRF, *γ*-tocotrienol, has been reported to decrease CCND1 gene expression [23].

Increased expression of miR-34a did not result in SIRT1 mRNA degradation even though translational inhibition of SIRT1 by miR-34a upregulation has been reported [43]. TRF treatment was found to increase SIRT1 gene expression directly without miR-34a modulation. Increased SIRT1 gene expression by TRF may compensate the reduction of this gene during aging [40] and hence increase the oxidative stress response.

Although previous study demonstrated that miR-34a is the direct transcriptional target of p53 [42], upregulation of miR-34a expression in this study was not accompanied with the increase of TP53 gene expression. However, increased transcriptional activity of p53 in senescent cells without elevated p53 gene and protein expression was reported [44]. Alternatively, upregulation of miR-34a in senescent HDFs may be independent of p53 and modulated by other transcription factor, such as ELK1 [45]. TRF increased TP53 gene expression directly in young nontransfected and transfected HDFs and senescent HDFs.

p53-miR-34a-SIRT1 positive feedback loop suggested that p53 induces miR-34a expression which suppresses SIRT1, increasing p53 activity [46]. However, this positive feedback loop was vague when miR-34a expression increases in senescent cells and by ectopic delivery at transcriptional level. TRF highlighted the positive feedback loop by increasing SIRT1 expression to enhance p53 deacetylation when miR-34a is overexpressed.

This study also observed the downregulation of miR-449a in senescent HDFs. Similarly, genome-wide analysis of miRNA expression revealed miR-449a was downregulated with age [18]. In contrary, increased miR-449a expression was reported in deep sequencing analysis [17]. However, miR-449a expression was found to be negatively associated with CCND1 expression [47]. Increased CCND1 observed in senescent HDFs may contribute to the downregulation of miR-449a in senescent HDFs observed in this study. Furthermore, the seed sequences of miR-449a are similar to that of miR-34a (UGGCAGUGU) [48], indicating similar target genes including CCND1 [46], CCNE2 [47], and CDK6 [49]. Increase of miR-34a expression with higher relative expression value (REV) suggested miR-34a may have a more important role than miR-449a during replicative senescence of HDFs.

TRF treatment was found to have increased miR-449a expression in both young and senescent HDFs, indicating that TRF modulated miR-449a expression but not specifically for senescent cells. Increased miR-449a expression in young and senescent cells may be accompanied with the elevated level of miR-449a transcription regulator, E2F1, to promote cell cycle progression [50].

In this study, the proposed mechanism which underlies TRF mediated regulation of miRNAs may be attributed to its radical-scavenging effect [51]. RNase III enzyme Dicer is responsible in the production of mature miRNAs. Its function is inhibited by multiple stresses including reactive oxygen species [52], which is normally accumulated during aging [12]. TRF is suggested to modulate miRNAs posttranscriptionally by alleviating the effect of stress on Dicer, therefore affecting miRNAs biogenesis and expression levels.

**Figure 5:**
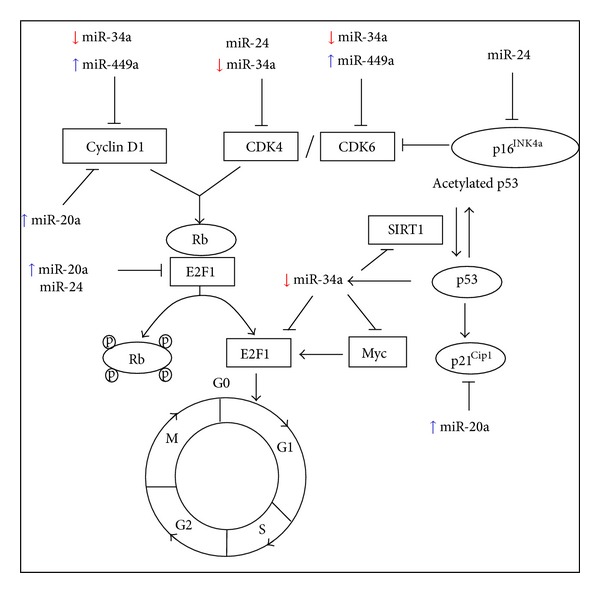
Modulatory effect of tocotrienol-rich fraction on the expression of SA-miRNAs at transcriptional level.

**Figure 6:**
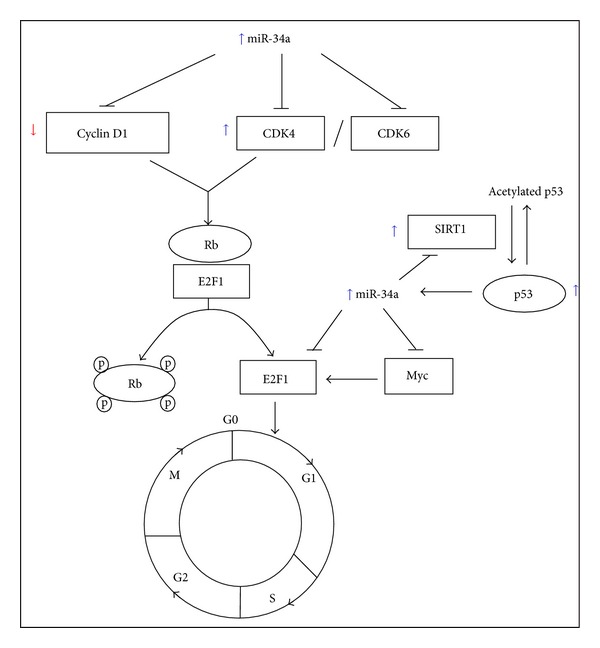
Modulatory effect of tocotrienol-rich fraction on the expression of miR-34a associated genes at transcriptional level when miR-34a is overexpressed.

Figure 5 summarized the modulatory effect of TRF on the expression of SA-miRNAs while Figure 6 summarized the modulatory effect of TRF on the expression of miR-34a associated genes when miR-34a is overexpressed. Our results revealed that TRF is a potential anticellular aging agent by modulating the expression of specific SA-miRNAs and its target genes involved in cell cycle regulation during cellular senescence.

## 5. Conclusion

In the present study, we demonstrated that tocotrienol-rich fraction with antioxidant and nonantioxidant properties altered the expression of SA-miRNAs specifically miR-34a and, therefore, alters the expression of miR-34a target genes involved in cell cycle regulation to promote cell cycle progression in senescent HDFs.

## Figures and Tables

**Table 1:** Forward primer sequences for validated miRNAs.

Accession number	miRBase ID	Mature miRNA sequences (5′→3′)	Size (bp)
miRBase			
MIMAT0000075	hsa-miR-20a-5p	UAAAGUGCUUAUAGUGCAGGUAG	23
MIMAT0000080	hsa-miR-24-3p	UGGCUCAGUUCAGCAGGAACAG	22
MIMAT0000255	hsa-miR-34a-5p	UGGCAGUGUCUUAGCUGGUUGU	22
MIMAT0004517	hsa-miR-106a-3p	CUGCAAUGUAAGCACUUCUUAC	22
MIMAT0001541	hsa-miR-449a	UGGCAGUGUAUUGUUAGCUGGU	22
NCBI			
NR_002752	RNU6B	CGCAAGGAUGACACGCAAAUUCGUGAAGCGUUCCAUAUUUUU	42

**Table 2:** Primers sequences for quantitative gene expression analysis.

Accession number	Gene	Primer	Primer sequences (5′→3′)	PCR product size (bp)
NM_002046	GAPDH	Forward	TCCCTGAGCTGAACGGGAAG	217
	GAPDH	Reverse	GGAGGAGTGGGTGTCGCTGT	

NM_053056	CCND1	Forward	AGACCTTCGTTGCCCTCTGT	181
	CCND1	Reverse	CAGTCCGGGTCACACTTGAT	

NM_000075	CDK4	Forward	TGGCCCTCAAGAGTGTGAGA	147
	CDK4	Reverse	ATGTGGCACAGACGTCCATC	

NM_012238	SIRT1	Forward	GCAGATTAGTAGGCGGCTTG	152
	SIRT1	Reverse	TCTGGCATGTCCCACTATCA	

NM_000546	TP53	Forward	GGAAGAGAATCTCCGCAAGAA	177
	TP53	Reverse	AGCTCTCGGAACATCTCGAAG	

## Abbreviations

AGE: Advanced glycosylation end product
AMPK: Adenosine monophosphate-activated protein kinase
CCND1: Cyclin D1
CCNE2: Cyclin E2
CDKI: Cyclin-dependent kinase inhibitor
CDK: Cyclin-dependent kinase
DMEM: Dulbecco's modified Eagle medium
ELK: ETS-like gene 1
E2F: E2 promoter binding factor
FBS: Foetal bovine serum
GADPH: Glyceraldehydes 3-phosphate dehydrogenase
HDF: Human diploid fibroblast
IGF-1: Inculin-like growth factor 1
miRNA: MicroRNA
PBS: Phosphate buffered saline
PD: Population doubling
pRb: Retinoblastoma protein
qRT-PCR: Quantitative reverse transcription-polymerase chain reaction
ROS: Reactive oxygen species
SA-miRNAs: Senescence-associated microRNAs
SA-*β*-gal: Senescence-associated beta-galactosidase
SIRT1: Sirtuin 1
TOR: Target of rapamycin
TP53: Tumour protein 53
TRF: Tocotrienol-rich fraction
UTR: Untranslated region.
